# Telephone communication on the ICU: What nurses say to families

**DOI:** 10.1186/2197-425X-3-S1-A713

**Published:** 2015-10-01

**Authors:** N Sangala, S Horscraft, A McGowan, F Baldwin

**Affiliations:** Royal Sussex County Hospital, Intensive Care Unit, Brighton, United Kingdom

## Introduction

Families of patients consider timely, clear, and compassionate communication as an indicator of quality care in the ICU(1). Good communication with the family members of patients dying on the ICU can reduce the burden of bereavement, whilst better communication throughout the admission can improve the family experience of ICU in general (2). Nursing staff are major contributors to family communication through their frequent interactions at the bedside. Nursing staff also provide support to family members over the telephone.

## Objectives

We performed a prospective observational study to understand the specific needs of family members during telephone calls with ICU nurses, and the ability of nurses to meet those needs.

## Methods

We developed a questionnaire that was completed by nursing staff who had received telephone calls from the family and friends of patients over a one week period. The questionnaire was specifically designed to identify the needs of the caller, and additionally record the time and length of the call, the accessibility of the nurses, and record any adverse events that occurred during the telephone conversation.

## Results

During the one-week period 66 questionnaires were returned of which 2 were incomplete and not used for analysis. Family members made the majority of calls with 31% coming from the patients' partner, 31% from their children, and 20% from other family members. Carers, police, alcohol liaison services and friends made the remaining calls. 21/64 calls were made out of hours yet 87% of callers reached the nurse at their first try. Conversations lasted between 2-10 minutes at an average of 4.3 minutes. The only adverse event reported was the loss of a single NG tube. The clinical condition of patients was the primary focus in 38% of calls followed by emotional support in 13% of calls. In a further 36% of calls, both clinical condition and emotional support were the predominant focus of the conversation. Non-clinical information dominated only 5 of 64 conversations and nurses rarely took time to gather information regarding their patient from family members (14/64). On 6 occasions the nursing staff were unable to answer all of the questions posed to them due to the requesting of detailed scan results (3/6), questions of prognostication (1/6), confidentiality issues (1/6), and a non-clinical explanation of visiting times (1/6).

## Conclusions

The predominant needs of family during telephone conversations with ICU nurses are the desire for clinical information and the need for emotional support. Nurses are readily accessible at all times of the day and provide a valuable source of information and support for family members over the telephone.
